# Labyrinth Metasurface for Biosensing Applications: Numerical Study on the New Paradigm of Metageometries

**DOI:** 10.3390/s19204396

**Published:** 2019-10-11

**Authors:** Irati Jáuregui-López, Pablo Rodríguez-Ulibarri, Sergei A. Kuznetsov, Carlos Quemada, Miguel Beruete

**Affiliations:** 1 Antennas Group-TERALAB, Universidad Pública de Navarra, Campus Arrosadía, 31006 Pamplona, Spain; irati.jauregui@unavarra.es (I.J.-L.); pabloru86@gmail.com (P.R.-U.); carlos.quemada@unavarra.es (C.Q.); 2 Multispectral Biosensing Group, Navarrabiomed, Complejo Hospitalario de Navarra (CHN), Universidad Pública de Navarra (UPNA), IdiSNA. Irunlarrea 3, 31008 Pamplona, Navarra, Spain; 3 Rzhanov Institute of Semiconductor Physics SB RAS, Novosibirsk Branch “TDIAM”, Lavrentiev Ave. 2/1, 630090 Novosibirsk, Russia; SAKuznetsov@nsm.nsu.ru; 4 Novosibirsk State University, Pirogova St. 2, 630090 Novosibirsk, Russia; 5 Institute of Smart Cities (ISC), Public University of Navarra, 31006 Pamplona, Spain

**Keywords:** metasurface, terahertz, biosensor

## Abstract

The use of metasurfaces operating in the terahertz regime as biosensor devices has attracted increased interest in recent years due to their enhanced sensitivity and more accurate detection capability. Typical designs are based on the replica of relatively simple unit cells, usually called metaatoms. In a previous paper, we proposed a new paradigm for ultrasensitive thin-film sensors based on complex unit cells, called generically metageometries or labyrinth metasurfaces. Here, we extend this concept towards biosensing, evaluating the performance of the labyrinth as a fungi detector. The sensing capabilities are numerically evaluated and a comparison with previous works in this field is performed, showing that metageometries improve the performance compared to metaatoms both in sensitivity and figure of merit, by a factor of more than four. In particular, we find that it is able to detect five fungi elements scattered on the unit cell, equivalent to a concentration of only 0.004/µm^2^.

## 1. Introduction

Due to its importance in human health as well as basic research, biological sensing, or biosensing, is gaining an increased interest amongst the scientific community. Biological sensing refers to the measurement of biological or chemical parameters and is based on the specific interaction between the entity under test (usually called analyte) and the sensing platform itself [1]. Most biomolecules have characteristic vibrational modes in the terahertz (THz) range (0.1–10 THz), which makes this band an excellent candidate for the development of new biosensing devices, or as an aid to complement and enrich the performance of existing ones [2]. Contrary to other traditional techniques, such as X-rays, THz radiation is non-ionizing and therefore harmless to living organisms. Some other advantages of THz waves are their sensitivity to water, making it suitable for tumor or cancer detection and diagnosis, since tumor cells contain a different water percentage than healthy cells [3]. For these reasons, THz waves have been investigated in a wide variety of biosensing applications, such as diseases diagnosis [4], DNA sequencing [5], detection of antibiotics [6] and protein concentrations [7], and detection of bacteria [8] and viruses [9], among others. 

Metamaterials can be defined as artificial materials engineered to present desired electromagnetic properties by controlling their shape and geometry. They are usually composed of an array of resonators much smaller than the operational wavelength. As a consequence of their reduced electrical size, they produce high electric field confinement at localized spots giving rise to strong changes in the spectral response, a property that can be exploited in sensing applications [1]. In the last years, they have been proposed to implement high quality biosensing devices. Metasurfaces—the planar version of metamaterials—have also emerged as an excellent alternative in the design of label-free sensing devices in biological applications [10]. They are very sensitive to changes in the refractive index near the structure, making possible a relatively cheap and easy way to detect samples deposited on the surface, with a great potential in sensitive label-free detection. In this way, metasurface sensors overcome the limitations of classical techniques [11], such as time-domain spectroscopy, where the major problem is the difference in sizes between the wavelength of the THz wave (~10–1000 µm), and the thickness of the sample to be analyzed (<1 µm), sometimes causing the sample to be invisible to the radiation. With the use of metasurfaces this problem is solved since sensing is based on the response of the structure itself, instead of measuring the sample response. As discussed in [12] there are other popular strategies for THz sensors, such as waveguides (both dielectric and metallic) and plasmonic structures. A crucial advantage of metasurface sensors is the flexibility they have to adapt to experimental setups, with direct wave illumination. This simplicity along with their enhanced sensitivity have put them in the vanguard of THz sensing applications. To date, metasurfaces operating in the THz regime have also shown promising results in microorganism biosensing, with designs capable of detecting very low concentrations of bacteria [8], yeast [13], and viruses [9,14].

Since their first conception, many different designs have been proposed, from the simplest designs [15,16], to more complex ones including graphene [17], or combination of slot antenna arrays with silver nanowires [14]. Whichever the structure employed, the main challenge in biosensing applications is how to achieve a high quality sensor when the analyte has an extremely small size compared to the wavelength, and hence it does not interact sufficiently with the radiating waves. The main goal of metasurface sensors is to provide high electric field confinement within the structure and enhance the detection of small analytes [10]. In order to address this challenge, a new paradigm shift from metaatoms (metasurfaces with discrete resonators) to more elaborated metageometries has been recently proposed [18]. Metageometries are designed to have the electric field highly confined all along the surface and not only at discrete points, which is a clear advantage for sensing applications compared to the “metaatom” approach. A further discussion comparing the performance of different metasurface sensor geometries at THz can be found in a recent review [10].

In a previous work [18], we demonstrated the excellent performance of metageometries in thin-film sensing applications by means of a labyrinth metasurface absorber working at THz. This structure is able to detect thin-films with a thickness of 10^−5^ times less than the operation wavelength, improving largely the results of metaatom-based sensors realized with the same manufacturing techniques. In this paper, we extend the scope of metageometries towards biosensing applications, performing a thorough numerical analysis of the labyrinth metasurface as a fungi sensor and compare its performance with previous works [8]. Our results demonstrate that the labyrinth metasurface is also promising in biosensing applications.

## 2. Materials and Methods

**Figure 1:**
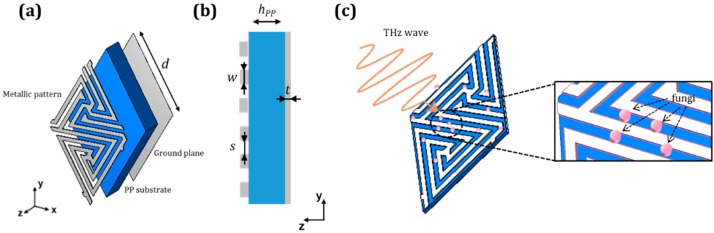
Front (**a**) and cross-sectional (**b**) views of the designed labyrinth metasurface unit cell. Metallization is shown in gray and polypropylene (PP) substrate in blue. Relevant dimensions: *h_PP_* = 29 µm, *d* = 36.4 µm, *t* = 0.4 µm, *s* = 1.5 µm; *w* = 1.5 µm. (**c**) Schematic of the labyrinth metasurface working as a fungi metasensor. Fungi are modeled as cylinders of radius *r_f_* = 2 µm, height *h_f_* = 1 µm, and dielectric permittivity *ε_f_* = 8 and are randomly distributed on the unit cell surface.

The metasurface sensor investigated in this work was designed to operate in absorption and was configured as a tri-layer structure with a metallic labyrinth pattern deposited on a flexible polypropylene (PP) slab 29 µm thick with back metallization (ground plane or GP), as shown in Figure 1. The geometry of the labyrinth pattern, which utilizes convoluted-shape apertures arranged on a triangular lattice, was taken from [18] and then downscaled by approximately 6.7 times to put the absorption resonance within the frequency range of 0.6–1 THz. The relevant metasurface dimensions are periodicity, *d* = 36.4 µm; distance between metallic strips, *s* = 1.5 µm; strip width *w* = 1.5 µm; metallization thickness *t* = 0.4 µm. In a practical implementation, the small-scale thin-film structure of this kind should be supported by a massive wafer (e.g., fabricated on top of a carrying silicon substrate). Evidently, thanks to the GP layer that blocks the transmission, the presence of the wafer would not affect the sensor’s performance.

**Figure 2:**
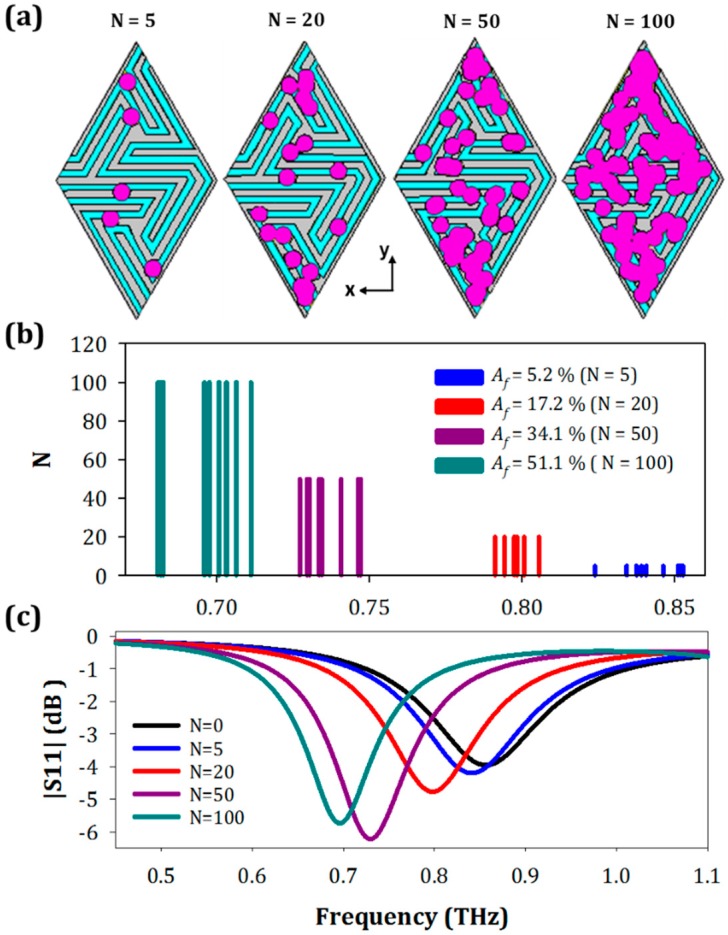
(**a**) Front view of the structure with *N* = 5; 20; 50; 100 fungi (purple cylinders). (**b**) Resonance frequency for all the simulations carried out for different fungi concentrations: *N* = 5 (blue curve), *N* = 20 (red curve), *N* = 50 (purple curve), and *N* = 100 (dark cyan curve). (**c**) Reflection coefficient for different fungi concentrations, cases nearest to the mean values.

The designed labyrinth metasurface was simulated in the band of 0.6–1 THz using the commercial simulator CST Microwave Studio®. To model the labyrinth metasurface as an infinite array, the regime of Floquet ports and periodic boundary conditions applied to the designed unit cell was employed. PP was modeled as a low-loss dielectric with a relative permittivity *ε_PP_* = 2.25·(1 − *j*⋅10^−3^) extrapolated from our previous study [18,19]. The metallic layers of the labyrinth pattern and GP were modeled as a lossy metal with electrical conductivity *σ* = 1.5 × 10^7^ S/m which mimics aluminum (Al) deposited on PP; as found in [20], this value is reduced compared to the nominal conductivity of bulk Al due to inherent surface roughness and granularities of PP films [19]. The excitation of the metasurface was done at normal incidence and vertical polarization, as shown in Figure 1c. It is worth noting that the labyrinth geometry is polarization insensitive under normal illumination [18]. The response of the analyte-free structure exhibits a narrow dip in the reflection coefficient at 0.856 THz (see the black curve in Figure 2c).

The performance of the labyrinth metasurface as a biosensor was evaluated numerically by placing fungi onto the patterned layer of the metasurface. The reason for using fungi as analyte is that their size, of the order of micrometers [8], is similar to the metasurface gaps, so they can be “trapped” in those gaps where high electric field confinement occurs, thereby enabling high sensitivity detection. Fungi were represented as dielectric cylinders with a radius *r_f_* = 2 µm, height *h_f_* = 1 µm and dielectric permittivity of *ε_f_* = 8, following the model obtained for yeast reported in [8]. Fungi elements were arbitrarily scattered within the unit cell using a randomizing function for the fungi position in both *x* and *y* axes, ensuring that all the cylinders fall within the cell limits. We considered possible fungi overlapping since this may occur in a real deposition scenario. Note that, due to computational restraints, in our approximation we are assuming the same fungi distribution for all unit cells of the structure, which is not completely realistic in practical sensing applications. An analysis of the whole structure would be preferable but extremely demanding in computation resources. The approximation done here gives a good hint about the performance of the labyrinth metasurface and allows for a sufficiently accurate analysis of its potential as biosensor. The deposition thresholds (i.e., number of cylinders deposited in each case) were chosen after a thorough study, selecting the cases to avoid overlap between the response of different concentrations of fungi.

## 3. Results and Discussion

As a first step, we performed a statistical numerical study in order to guarantee that the results have adequate repeatability. The number of fungi was varied from *N* = 5 to *N* = 100 elements and 10 different simulations were done in each case. A schematic of the fungi location over the surface is shown in Figure 2. For the sake of clarity, only the four most significant results are presented in Figure 2, *N* = 5, 20, 50, and 100. Note that our main objective is to find the minimal amount of sample detectable by the proposed metasensor. 

As can be observed in Figure 2b, for an identical number of fungi the resonance frequency experiences slight variations depending on the fungi position within the unit cell. This is explained by a non-uniform electric field distribution over the metasurface unit cell. Despite these variations, the detection thresholds were chosen to avoid overlap between consecutive cases allowing for an unambiguous detection. To get a better description of the metasurface performance, the results of Figure 2b were represented in terms of a fractional area defined as *A_f_ = A_o_/A_T_*, where *A_o_* represents the area occupied by the microorganisms (extracted directly from the simulator), and *A_T_* the total area of the metasensor surface (calculated analytically). Since *A*_0_ has some variations, depending on the fungi overlap, we took an average of all cases, obtaining mean fractional areas of 5.23% (*N* = 5), 17.2% (*N* = 20), 34.1% (*N* = 50), and 51.13% (*N* = 100). 

The spectral performance of the labyrinth metasensor can be grasped from the selected representative curves of Figure 2c. To select these curves, we calculated in the first place the mean value of the resonance frequency for each *N*. Afterwards, we looked for the simulation case whose resonance frequency was closer to the calculated mean resonance frequency value. These are the curves represented in the figure. From the simulation results of Figure 2b,c, we find that the maximum resonance frequency shift happens for *N* = 100, with a variation of nearly 19% (from 856 to 696 GHz) and a standard deviation of 10.3 GHz. The minimum is obtained for *N* = 5, with a resonance frequency shift of 1.6% and a standard deviation of 8.5 GHz. These results suggest that the metasensor designed is able to discriminate small concentrations of microorganisms deposited on top.

In order to characterize quantitatively the performance of the structure, we calculated its sensitivity (S) and figure of merit (FOM). Although there are many different definitions for the sensitivity in the literature, see [10,21], almost all of them link the resonance frequency shift with some other parameter that indicates the quantity of sample deposited on the metasensor. Thus, in this work we defined the sensitivity as S = Δ*f*/*A_f_·n* where Δ*f* = *f_N_* − *f*_0_, with *f_N_* the resonance frequency for each fungi concentration, *N*, and *f*_0_ the resonance frequency without the analyte; *A_f_* the fractional area occupied by the fungi; and *n* the refractive index of the fungi. The FOM is a more refined parameter defined as the ratio between the sensitivity and the full width at half minimum (FWHM) in frequency dimensions: FOM = S/FWHM, which takes into account the spectral linewidth of different curves, making structures with a small FWHM more appropriate for biosensing applications, as in that case, the discrepancy between different curves becomes easier to detect. Thus, a narrower spectral line will lead to higher FOM values. With these definitions, we obtained a mean sensitivity of 80 GHz/RIU, and a mean FOM of 0.85 (RIU)^−1^, where RIU stands for refractive index units (RIU). All the values of fractional area, resonance frequency, sensitivity, and FOM for each fungi concentration are summarized in Table 1, at the end of this section. The remaining results and specific values for every simulated case are presented in the Supplementary Material.

To evaluate more deeply the labyrinth metasensor, we compared its performance with other sensors found in the literature. We chose for this comparison the work presented in [8], which is one of the most significant in terms of microorganism detection using metasurfaces, due to the high sensitivity values achieved. Therein, a metaatom-based metasurface made of a square ring with a gap at the center unit cell was used to detect different types of fungi. Microorganisms were deposited arbitrarily at a minimal density of 0.09/µm^2^ (calculated as the ratio between the average number of microorganisms in the capacitive gap of the metasurface, and the gap area) and showed a displacement of the resonant frequency of 9 GHz (corresponding to 1% of the resonant frequency of the bare structure). In our case, we arbitrarily deposited fungi elements at a minimal density of 0.004/µm^2^ (corresponding to *N* = 5 fungi and calculated as the ratio between the fungi elements and the total surface area), and we obtained a frequency shift of 14 GHz (1.6% of the resonant frequency). This means a large improvement of the previous work, as we get a larger shift with a much lower density. 

**Figure 3:**
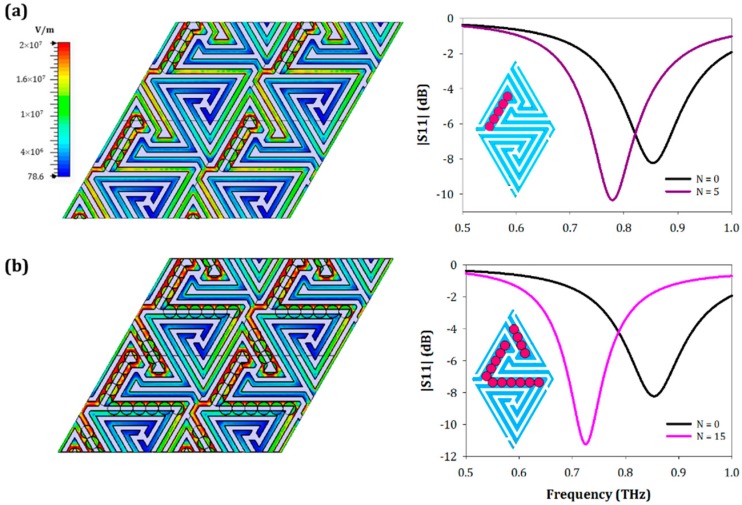
(**a**) Electric field distribution over the labyrinth surface for the designed structure with *N* = 5 (*A_f_* = 5.48%) fungi placed at the regions of maximum electric field confinement (left) and reflection coefficient comparison with the bare structure (right). (**b**) Electric field distribution (left) and reflection coefficient (right) when placing N = 15 (*A_f_* = 16.45%) fungi elements in the places where the electric field confinement is maximum (pink curve), and comparison with the bare structure (black curve).

To find the maximum performance of the designed structure in [8], *N* = 5 fungi elements were deposited in the micro-gap of the metasurface, where the electric field concentration is maximum. In that case, Park et al. [8] achieved a frequency shift of 2% (~15 GHz). Applying a similar procedure to the labyrinth metasurface, we find that with the deposition of *N* = 5 fungi elements in the area of maximum electric field magnitude (see Figure 3a), a frequency shift of 9% (77 GHz) is obtained, improving the previous results by a factor of more than 4. 

This excellent performance is due to the intricate geometry of the metasensor, which provokes a strong electric field confinement between the adjacent metallic strips of the surface, scattered within the unit cell area and not concentrated at discrete spots. In fact, as it can be seen in Figure 3, there are more regions with high electric field confinement. If we cover systematically all these zones with *N* = 15 fungi elements, as shown in Figure 3b, the frequency shift of the resonance is much larger. Thus, with only 15 fungi elements, which represent a fractional area of 16.45%, we obtain a frequency shift of 134 GHz similar to the case of *N* = 100 fungi elements analyzed above, demonstrating the importance of the element’s location. We obtained sensitivity and FOM values that are well above those obtained with a much larger number of randomly deposited fungi elements. Concretely, we reached a maximum sensitivity of 351.9 GHz/RIU, and a maximum FOM of 2 (RIU)^−1^ for the case of coating the surface with *N* = 5 fungi placed in the microgap of the surface. If we look at the comparison described in Table 1, we can verify that the values for this case almost triple those obtained in the rest of the study. It is also noticed that the sensitivity and FOM values are greater for the case of *N* = 5 fungi instead of *N* = 15 fungi ordered in the area of interest. Although the frequency shift is larger in the second case, the fractional area occupied is also higher, causing a decrease in the sensitivity.

## 5. Conclusions

To conclude, we reported here a labyrinth metasurface operating at THz with high sensitivity and FOM working as a fungi biosensor. The use of convoluted metageometries instead of metaatoms improves the electric field concentration within the surface and not only at discrete points. The behavior of the structure was numerically tested obtaining a mean sensitivity of 80 GHz/RIU, and a mean FOM of 0.85 (RIU)^−1^ when depositing different fungi concentrations, from *N* = 5 to *N* = 100 arbitrarily at random positions. Furthermore, a study of the electric field distribution over the metasensor surface was done. By identifying the regions of high electric field concentration, it was determined that with a number of fungi elements as low as *N* = 5, a high frequency shift of 9% (77 GHz) is obtained, improving previous works based on metaatoms by a factor of more than 4. Moreover, depositing *N* = 15 fungi along all the area where the electric field distribution is maximum, a frequency shift of 134 GHz is achieved, similar to the case of having *N* = 100 fungi elements at random positions. This highlights the importance of the fungi location in the device and the better performance of metageometry-based designs in comparison with classical structures utilizing metaatoms. These new structures, where the enhanced electric field is distributed over a much larger surface area of the device, can be of high interest for biological sensing when the amount of sample under measurement is relatively small.

The study presented in this work is limited to numerical investigations. Fabricating the proposed metasurface biosensor and its experimental testing is under consideration now. The technological aspects of the sensor’s fabrication, as well as the issues of sensor’s viability and contamination of the substrate, are beyond the scope of this paper and will be published elsewhere.

## Figures and Tables

**Table 1:** Comparison of the most relevant performance parameters of the labyrinth metasurface structure, for different numbers of fungi deposited on top. The cases highlighted with (*) correspond to fungi placed in the regions of maximum field intensity.

*N*	Fractional Area (%)	Δ*f* (GHz)	Δ*f* (%)	Standard Deviation	Sensitivity (GHz/RIU)	FOM (RIU)^−1^
0	0	856	-	-	-	-
5	5.23	842	1.64	8.5	67.3	0.73
20	17.21	797	6.85	5	85.2	0.89
50	34.08	736	13.94	7.5	87.6	0.92
100	51.13	696	18.68	10.3	78.2	0.87
5 *	5.48	778	9.02	-	351.9	2
15 *	16.45	722	15.64	-	203.4	1.36

## Supplementary Materials

The following are available online at https://www.mdpi.com/1424-8220/19/20/4396/s1, Table S1: Frequency resonance for all the simulations carried out for each fungi concentration, average and typical deviation for each case.
