# Supplementary material for: Labyrinth Metasurface for Biosensing Applications: Numerical Study on the New Paradigm of Metageometries
Source: Sensors (Basel). 2019 Oct 11;19(20):4396. doi: 10.3390/s19204396 (PMC6833032; doi:10.3390/s19204396)
Supplement: Supplementary file 1 [file sensors-19-04396-s001.pdf]

sensors

MDPI

Labyrinth metasurface for biosensing applications: the new paradigm of metageometries

Irati Jáuregui-López[1, 2], Pablo Rodriguez-Ulibarri[1], and Miguel Beruete[1, 2,*]

[1] Antennas Group-TERALAB, Universidad Pública de Navarra, Campus Arrosadía, 31006 Pamplona, Spain; irati.jauregui@unavarra.es
[2] Multispectral Biosensing Group, Navarrabiomed, Complejo Hospitalario de Navarra (CHN), Universidad Pública de Navarra (UPNA), IdiSNA. Irunlarrea 3, 31008 Pamplona, Navarra, Spain
* Correspondence: miguel.beruete@unavarra.es; Tel.: +34-948-169-727

Received: 13 September 2019; Accepted: 9 October 2019; Published: 11 October 2019

Supplementary materials

Table S1. Frequency resonance for all the simulations carried out for each fungi concentration, average and typical deviation for each case

N	Frequency resonance for different simulations (GHz)										Avg.	Typical deviation
0	856	856	856	856	856	856	856	856	856	856		
5	846.4	837.6	851.2	840.8	840.8	839.2	834.4	852	852.8	824	841.92	8.55
20	794.4	805.6	794.4	800.8	794.4	798.4	791.2	797.6	805.6	791.2	797.36	5
50	729.6	746.4	734.4	747.2	733.6	729.6	727.2	740.8	730.4	747.2	736.64	7.59
100	697.6	680.8	700.8	682.4	706.4	711.2	696	703.2	681.6	700.8	696.08	10.31
